# Characterization of H7N2 Avian Influenza Virus in Wild Birds and Pikas in Qinghai-Tibet Plateau Area

**DOI:** 10.1038/srep30974

**Published:** 2016-08-24

**Authors:** Shuo Su, Gang Xing, Junhua Wang, Zengkui Li, Jinyan Gu, Liping Yan, Jing Lei, Senlin Ji, Boli Hu, Gregory C. Gray, Yan Yan, Jiyong Zhou

**Affiliations:** 1 Jiangsu Engineering Laboratory of Animal Immunology, Institute of Immunology and College of Veterinary Medicine, Nanjing Agricultural University, Nanjing, China; 2 Key Laboratory of Animal Virology of Ministry of Agriculture, College of Animal Sciences, Zhejiang University, Hangzhou, China; 3 College of Agriculture and Animal Husbandry, Qinghai University, Xining, China; 4 Division of Infectious Diseases, Global Health Institute, & Nicholas School of the Environment, Duke University, Durham, NC, USA; 5 Collaborative Innovation Center and State Key Laboratory for Diagnosis and Treatment of Infectious Diseases, The First Affiliated Hospital, Zhejiang University, Hangzhou, China

## Abstract

Qinghai Lake is a major migrating bird breeding site that has experienced several recent highly pathogenic avian influenza virus (HPAIV) epizootics. From 2006 to 2009 we studied Qinghai’s wild birds and pikas for evidence of AIV infections. We sampled 941 healthy wild animals and isolated seventeen H7N2 viruses (eight from pikas and nine from wild birds). The H7N2 viruses were phylogenetically closely related to each other and to viruses isolated in Hong Kong in the 1970s. We determined the pathogenicity of the H7N2 viruses by infecting chickens and mice. Our results suggest that pikas might play an important role in the ecology of AIVs, acting as intermediate hosts in which viruses become more adapted to mammals. Our findings of AI infection in pikas are consistent with previous observations and raise the possibility that pikas might play a previously unrecognized role in the ecology of AIVs peridomestic aquatic environments.

Influenza A viruses (IAVs) exhibit a wide host range that includes birds and mammals. Viruses exhibit varying degrees of host adaptation after crossing species barriers^1^,^2^. Influenza A viruses pose a continual threat to humans and animals because they frequently change via mutation and/or reassortment.

Wild aquatic birds are the main natural reservoir of IAVs, harboring 16 of 18 hemagglutinin (HA) and 9 of 11 recognized neuraminidase (NAs) glycoproteins^3^,^4^,^5^,^6^. IAVs replicate in the gastrointestinal track of aquatic birds and are most often spread through the fecal-orally route^2^. They generally cause little or no signs of disease in birds. Migratory birds have a the potential to spread these viruses over large geographical areas.

Qinghai Lake, the largest lake in China is located in the Qinghai-Tibet plateau and serves as a home for ~150,000 birds each year. It constitutes major stop area for various migration flyways. Notably, after highly pathogenic avian influenza (HPAI) H5N1 outbreaks occurred among wild birds at Qinghai Lake in 2005 and 2006^7^,^8^, HPAI H5N1 strains spread across major flyways to North America, Eurasia, and Africa^9^ suggesting a geotemporal association.

The pika (*Ochotona curzoniae*), a small rabbit-like mammal, is a natural resident of the Qinghai-Tibet plateau that has recently been considered as a natural host for influenza viruses^10^,^11^. Sialic acids with affinity for human and avian IAVs are expressed in the respiratory tract of pikas^12^. Previously, HPAI H5N1 viruses have been isolated from pikas^10^ and pikas have been shown to be seropositive against H9N2 low pathogenic avian influenza (LPAI) viruses^11^. Additionally, experimental infections have shown that pikas are susceptible to HPAI H5N1, LPAI H9N2, human H1N1, and human H3N2 viruses^12^. These data suggest that pikas could play an important role in influenza ecology, and thus might deserve more attention in surveillance studies.

In the past, surveillance for influenza viruses in Qinghai Province focused mainly on aquatic birds and poultry^7^,^8^,^13^,^14^,^15^,^16^,^17^ and wild mammals have seldom been studied. With this knowledge gap in mind, we conducted this study of wild birds and pikas.

## Materials and Methods

### Sample collection

**Figure 1:**
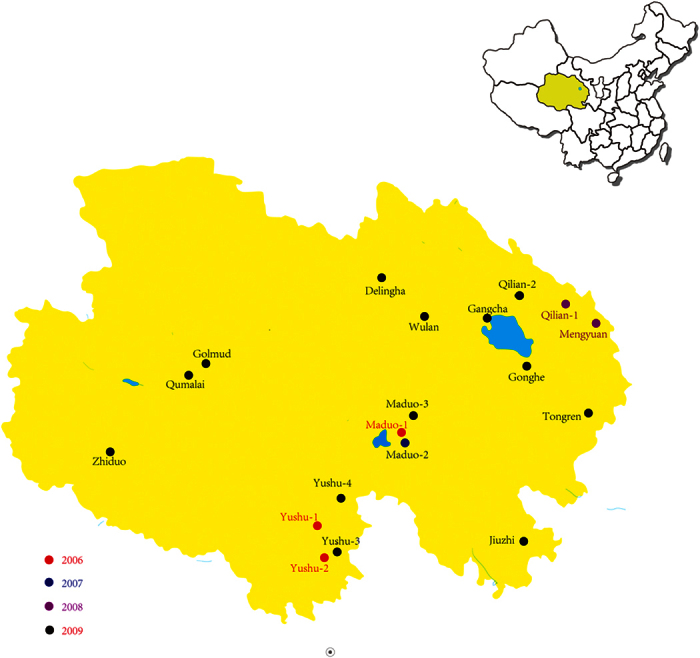
The sampling sites in Qinghai Province, China. The circles indicate dates and locations of animal-trapping sites. The picture is created by Photoshop CS5.

In an effort to evaluate wild animals in the Qinghai Province ecosystem, we captured wild birds and black-lipped pikas (*Ochotona curzoniae*) from August 2006 to October 2009 in Qinghai Province some captured site where are on the distributions of generalized migration routes of migratory birds^18^). Specific sites included most areas around the Qinghai lake: Qumalai, Zhiduo, Jiuzhi, Maduo, Golmud, Delingha, Wulan, Gangsha, Gonghe, Tongren, Qilian, Mengyuan and Yushu (Fig. 1). All captured animals were clinically healthy. Aquatic and passerine birds were swabbed and released. Pikas were euthanized and their tissues (heart, liver, spleen, lungs, kidneys, and brain) were harvested and preserved in liquid nitrogen.

### Virus isolation and identification

Virus isolation in 10-day-old specific pathogen free (SPF) embryonated chicken eggs was attempted from each tissue sample. Virus subtyping was performed by RT-PCR^19^.

### Genome sequencing and phylogenetic tree analysis

Viruses were sequenced as previously described^10^. Briefly, total RNA was extracted from allantoic fluid with Trizol^®^ LS (Invitrogen, Carlsbad, CA), followed by RT-PCR using a One-Step RT-PCR kit (Qiagen, Hilden, Germany) with universal influenza primers^20^. PCR products were cloned into the pMD18-T vector, and sequenced. Viral sequences were aligned using Clustal W within the BioEdit software package (version 5.0.9). Maximum likelihood phylogenetic trees were inferred with 1000 bootstraps using MEGA (Version 6).

### Animal infections

We characterized the *in vivo* pathogenicity of two AIVs isolated from pikas: A/Pika/QH-Maduo/01/2006 (Maduo06) and A/Pika/Maduo/01/2009(Maduo09). Ten 5-week-old SPF white leghorn chickens (obtained from the Merial Vital Laboratory Animal Technology Co. Ltd., Beijing, China) were housed in negative-pressure isolator cages with HEPA-filtered air. They were inoculated intravenously (i.v.) with 0.2 ml/chicken of a 1:10 dilution of bacteria-free fresh allantoic fluid containing a virus isolate. The process was performed according to OIE methods^21^ in evaluating the intravenous pathogenicity index (IVPI).

In addition, ten chickens were inoculated intranasally with 10^6.0^EID_50_/animal of each the viruses under study in a 0.2 ml volume, and 5 chickens were inoculated intranasally with 0.2 ml PBS as non-infection control. To evaluate bird to bird transmission, two additional chickens were housed with the inoculated chickens at 12 hours post infection. Clinical symptoms were recorded every day for 14 days. Oropharyngeal and cloacal swabs were collected from animals on days three, five, and seven post-inoculation (pi.) to evaluate viral shedding. Three chickens from each group were euthanized on day three pi to measure virus replication in lungs, intestine, pancreas, heart, spleen, kidney, thymus, and brain. Viral titers (EID_50_) were calculated using the method of Reed and Muench^22^.

Six-week-old SPF BALB/c mice obtained from the Experiment Animal Center of Zhejiang University, Hangzhou, China, were randomly divided into four groups (20/group). Mice were anesthetized with dry ice, and inoculated intranasally (i.n.) with 10^6.0^EID_50_ of Maduo06 and Maduo09 viruses in 50 μl PBS, respectively. As non-infection control, the other group (20 mice) were inoculated i.n. with 50 μl PBS. Mice were monitored daily for weight loss and mortality. Three mice in each group were euthanized on days three, five and seven pi to evaluate virus replication and histopathological changes in lung, intestine, pancreas, heart, spleen, kidney, thymus, and brain. The viral titers (EID_50_) were calculated using the method of Reed and Muench^22^.

### Ethics statement

Animal experiments were executed in accordance with the Regulations for the Administration of Affairs Concerning Experimental Animals approved by the State Council of the People’s Republic of China. All the animal experiments and samples collected were approved by the Institutional Animal Care and Use Committee (IACUC) of Zhejiang University, permission number: SYXK 2012-0178.

## Results

### Influenza isolation from the wild animals in Qinghai

From August 2006 to October 2009, 360 pikas, 74 resident wild birds, 14 migrating birds and 493 water birds were captured from thirteen sites of the Qinghai-Tibet plateau and a total of 22 viruses (2.3%) were isolated. HA and NA sequencing revealed that seventeen viruses were of the H7N2 subtype and five viruses were of the H5N1 type (H5N1 viruses were published before). For the H7N2 viruses (Table 1), two viruses were isolated from migrating birds (*Acanthis*), and seven viruses were isolated from resident birds (*Passer montanus*, *Melanocorypha mongolica*, *Podoces* and *Montifringilla*), and eight viruses were isolated from pikas.

### Molecular characterization and phylogenetic analysis of the H7N2 influenza viruses

**Figure 2:**
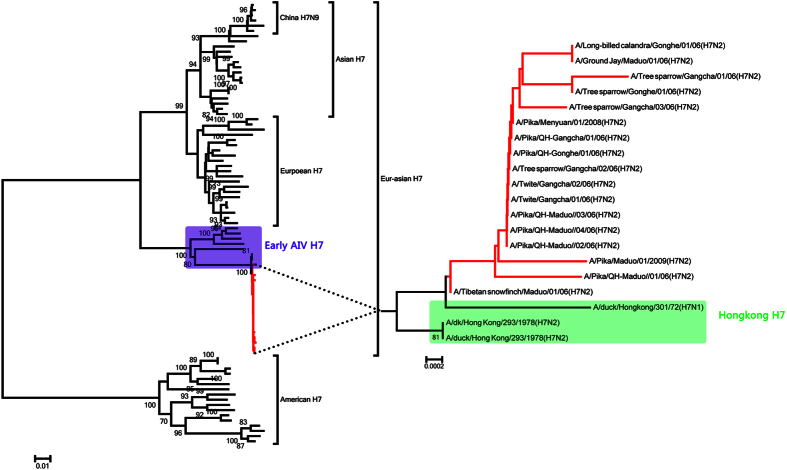
Phylogenetic analysis of the HA gene of the H7N2 isolates. Viruses isolated in this study are indicated in red. Maximum likelihood phylogenetic trees were inferred and with 1000 times bootstrap using MEGA (Version 7).

Phylogenetic analysis showed that the HA genes of all H7 subtype isolates were divided into two geographical distinct lineages (Eurasian and American) (Fig. 2). These individual groups were classified by temporal and geographic relationships rather than by host species. All H7N2 isolates were closely related to 1970s-era Hong Kong AI H7 isolates and clustered in a group, which belongs to the Eurasian branch (Fig. 2). These seventeen H7N2 viruses were very different by HA gene analyses from H7N9 viruses that have infected humans in China since 2013 (Fig. 2). Phylogenetic analysis of the NA and internal genes also showed clustering with H7 AIVs isolated in Hong Kong (Figure S1), suggesting that the H7N2 viruses isolated from pikas were of avian origin. We did not detect reassortment in any of H7N2 viruses.

We further examined the isolates for the presence of putative mammalian adaptive mutations and antiviral resistance (Table 2). The HA cleavage site of all H7N2 isolates is PEIPKGR, and the receptor binding sites in HA exhibited Q and G in positions 222 and 224, respectively (H3 numbering). No NA deletion was observed in any of the H7N2 isolates. Positions 627 and 701 in PB2 displayed E and D, respectively. Mutations in NA (Oseltamivir resistant amino acid sites: 119E, 274H and 292R) and M2 (Amantadine resistant amino acid sites: 26L, 27V, 30A and 31S) associated with antiviral resistance were not detected.

### Animal infections

**Figure 3:**
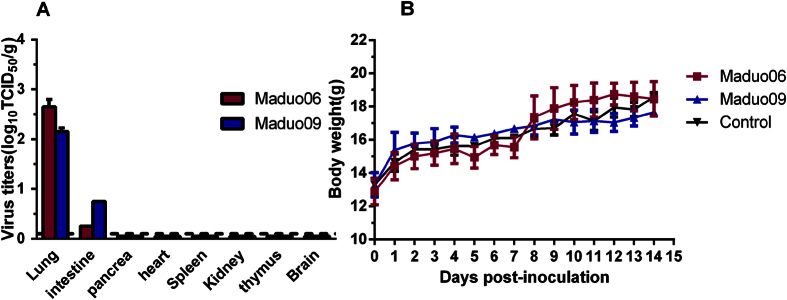
Viral replication in tissues of inoculated chickens (**A**) and the body weight of inoculation mice (**B**). Maduo06: A/Pika/QH-Maduo/01/2006(H7N2); Maduo09: A/Pika/Maduo/01/2009(H7N2).

Maduo06 and Maduo09 viruses were used to assess the pathogenicity of H7N2 viruses moving from pikas to chickens and mice. Results from pathogenicity studies indicated that the IVPI values in chickens were 0.17 (Maduo06) and 0.19 (Maduo09). Contact chickens showed no clinical symptoms, and the infected chickens showed unapparent clinical symptoms except for transient depression. High viral replication was only detected in lung tissues with a mean viral load titer of 2.65 ± 0.15 (log_10_EID_50_/g) for Maduo06 and 2.15 ± 0.08 (log_10_EID_50_/g) for Maduo09 (Fig. 3A). Low viral replication was detected in the intestines, with mean titers of 0.25 ± 0.00 (log_10_EID_50_/g) for Maduo06 and 0.75 ± 0.00 (log_10_EID_50_/g) for Maduo09 (Fig. 3A). Virus replication was not detected in other organs. Viruses were detected from the oropharynx and cloaca of infected chickens with maximum viral shedding occurring at seven dpi. Virus titers were between 1.25 ± 0.00 to 3.44 ± 0.58 (log_10_EID_50_/ml) (Table 3).

Mice survived the experimental infections with H7N2 viruses. Body weight increased over time and there were no differences when compared to the control group (Fig. 3B). No viral replication was detected in any of the organs of the euthanized mice. Histological examination revealed no pathological changes in animals infected with either virus. These data confirmed that the H7N2 viruses isolated from pikas were of low pathogenicity in chickens and mice.

## Discussion

Previous reports have suggested that plateau pikas can be asymptomatically infected with HPAI H5N1^10^ and LPAI H9N2^11^. The findings raise the possibility that pikas might play a role in the transmission of AIVs at Qinghai-Tibet Plateau and provide opportunities for the adaptation of AIVs to mammals. Our results provide evidence that H7N2 AIV has become enzootic in wild animals around Qinghai Lake. We isolated five H5N1^10^ and eight H7N2 viruses from pikas. Additionally, nine H7N2 viruses were isolated from wild birds. Overall, our findings suggest that both H5N1 and H7N2 viruses continue to circulate in the Qinghai-Tibet Plateau area ecosystem.

Our study validates a previous report suggesting that the wild pikas could serve as a previously unrecognized natural host of IAVs. Like pigs, pikas possess both avian and mammalian receptors and could potentially serve as “mixing vessels” for the generation of novel IAVs^12^. Of note, pikas have been experimentally infected with both human H1N1 and human H3N2 influenza viruses^12^. Their susceptibility to various influenza A viruses adds to the complex ecosystem around the Qinghai Lake in Qinghai-Tibet Plateau area. As species of wild birds may live in close contact with pikas in a den, we speculate that complex viral transmission patterns involving multiple species might commonly occur in this ecosystem.

From the animal experiments, we found that H7N2 isolates were low pathogenic to chickens and mice. In our field investigations, all samples were obtained from healthy-appearing wild animals. Under experimental conditions, the pika-associated H5N1viruses (2.2 clade and 2.3.2 clade) resulted in sub-clinical infection in rabbits (a species closely related to pikas)^10^. Hence, it seems possible that pikas may serve as healthy mammalian reservoir for IAVs. What is not clear is how to implement interventions that would reduce mammal contact with migrating aquatic birds or humans. The H5N1/H7N2/H9N2 AIVs infections in pikas detected here and past experiments suggests that they should be considered in future AIV surveillance programs at least for the Qinghai-Tibet Plateau area.

As mentioned above, Qinghai Lake is a major breeding site for migratory birds that overwinter from Southeast Asia, India, and Tibet and the migratory range of these birds is significant. In 2005, a HPAIV H5N1 outbreak occurred among migratory wild birds at Qinghai Lake^7^ threatening nearby poultry and mammalian species^17^. This H5N1 lineage subsequently spread throughout North America, Eurasia, and Africa^9^. This threat is likely to continue as multiple HPAI H5N1 lineages have been persistently circulating in Qinghai^8^,^14^,^15^,^16^ and H9N2 AIVs have also infected wild birds at Qinghai Lake^13^,^23^.

Asymptomatic infection of plateau pikas with H5, H7 and H9 subtype AIVs could lead to mammalian adaptation. Fortunately, in our study, the HA and NA genes of the H7N2 isolates had no evidence of recent reassortment, suggesting that these viruses have become enzootic and stable in these wild animals. It is unclear why the Qinghai H7N2 viruses which have the ability to infect both wild birds and mammals have not yet been detected in domestic poultry in the Qinghai-Tibet plateau area. Perhaps this is due to limited AIV surveillance among poultry in this area. It seems logical that if AIVs are enzootic among the many wild birds in the Qinghai-Tibet plateau area, especially around the Qinghai Lake area, infections in other wild and domestic animals are likely occurring but unrecognized. More extensive AIV surveillance among Qinghai Lakes migrating birds, wild mammals, and domestic animals seems imperative.

Our study highlights once again the importance of Qinghai-Tibet Plateau area ecosystem in influenza virus circulation. The finding of H7N2 subtype influenza viruses from wild birds and pikas expands the complexity of the influenza virus gene pool in Qinghai Province.

## Additional Information

**How to cite this article**: Su, S. *et al*. Characterization of H7N2 Avian Influenza Virus in Wild Birds and Pikas in Qinghai-Tibet Plateau Area. *Sci. Rep.* **6**, 30974; doi: 10.1038/srep30974 (2016).

## Supplementary Material

Supplementary Information

## Figures and Tables

**Table 1:** Characteristic of influenza A virus H7N2 isolates.

No.	Isolation time	Viruses name	Genus	Genebank Accession number (HA, NA, NS, M, NP, PA, PB2, PB1)
1	Aug. 2006	A/Twite/Gangcha/01/2006	*Acanthis*	KX130801, KX130821, KX156630, KX156647, KX156664, KX156681, KX156698, KX156715
2	Aug. 2006	A/Twite/Gangcha/02/2006	*Acanthis*	KX130802, KX130822, KX156631, KX156648, KX156665, KX156682, KX156699, KX156716
3	Aug. 2006	A/Tree sparrow/Gangcha/02/2006	*Passer montanus*	KX130803, KX130823, KX156632, KX156649, KX156666, KX156683, KX156700, KX156717
4	Aug. 2006	A/Tibetan snowfinch/Maduo/01/2006	*Passer montanus*	KX130804, KX130824, KX156633, KX156650, KX156667, KX156684, KX156701, KX156718
5	Aug. 2006	A/Tree sparrow/Gangcha/03/2006	*Passer montanus*	KX130805, KX130825, KX156634, KX156651, KX156668, KX156685, KX156702, KX156719
6	Aug. 2006	A/Tree sparrow/Gonghe/01/2006	*Passer montanus*	KX130806, KX130826, KX156635, KX156652, KX156669, KX156686, KX156703, KX156720
7	Aug. 2006	A/Long-billed calandra/Gonghe/01/2006	*Mongolian lark*	KX130807, KX130827, KX156636, KX156653, KX156670, KX156687, KX156704, KX156721
8	Aug. 2006	A/Tree sparrow/Gangcha/01/2006	*Podoces*	KX130808, KX130828, KX156637, KX156654, KX156671, KX156688, KX156705, KX156722
9	Aug. 2006	A/Ground Jay/Maduo/01/2006	*Montifringilla*	KX130809, KX130829, KX156638, KX156655, KX156672, KX156689, KX156706, KX156723
10	Aug. 2006	A/Pika/QH-Maduo/01/2006	*Ochotona curzoniae*	KX130810, KX130830, KX156639, KX156656, KX156673, KX156690, KX156707, KX156724
11	Aug. 2006	A/Pika/QH-Maduo/02/2006	*Ochotona curzoniae*	KX130811, KX130831, KX156640, KX156657, KX156674, KX156691, KX156708, KX156725
12	Aug. 2006	A/Pika/QH-Maduo/03/2006	*Ochotona curzoniae*	KX130812, KX130832, KX156641, KX156658, KX156675, KX156692, KX156709, KX156726
13	Aug. 2006	A/Pika/QH-Maduo/04/2006	*Ochotona curzoniae*	KX130813, KX130833, KX156642, KX156659, KX156676, KX156693, KX156710, KX156727
14	Aug. 2006	A/Pika/QH-Gonghe/01/2006	*Ochotona curzoniae*	KX130814, KX130834, KX156643, KX156660, KX156677, KX156694, KX156711, KX156728
15	Aug. 2006	A/Pika/QH-Gangcha/01/2006	*Ochotona curzoniae*	KX130815, KX130835, KX156644, KX156661, KX156678, KX156695, KX156712, KX156729
16	Nov. 2008	A/Pika/Menyuan/01/2008	*Ochotona curzoniae*	KX130819, KX130839, KX156645, KX156662, KX156679, KX156696, KX156713, KX156730
17	Apr. 2009	A/Pika/Maduo/01/2009	*Ochotona curzoniae*	KX130820, KX130840, KX156646, KX156663, KX156680, KX156697, KX156714, KX156731

**Table 2:** Molecular characterization of the HA, NA, M2, NS1, and PB2 gene sequences of the H7N2 isolates and other virus strains.

Virus strains	HA			NA	M2				NS1			PB2	
	Cleavagesite	Receptor binding sites		Stalk deletion	Amantadine resistant aa				5-aa deletion	Virulence determinant		Virulence determinant	
		222‡	224		26	27	30	31	80–84	92	PBM	627	701
17 H7N2 viruses*	PEIPKGR	Q	G	No	L	V	A	S	No	E	ESEV	E	D
Swine/KU/16/2001(H7N2)	PEIPKGR	Q	G	No	L	V	A	S	No	E	ESEV	E	D
Chicken/Hebei/1/2002(H7N2)	PEIPKGR	Q	G	No	L	V	A	S	No	E	ESEV	E	D
ML/Netherlands/29/06(H7N2)	PEIPKGR	Q	G	No	L	V	A	S	No	E	ESEV	E	D
Quail/Italy/4610/2003 (H7N2)	PEIPKGR	Q	G	No	L	V	A	S	No	E	ESEV	E	D
Duck/Tasmania/277/2007 (H7N2)	PEIPKGR	Q	G	No	L	V	A	S	No	E	ESEV	E	D
Chicken/NJ/118878-5/2001 (H7N2)	PEIPKGR	Q	G	No	L	V	A	S	No	E	ESEV	E	D
Chicken/NJ/16224-6/1999(H7N2)	PEIPKGR	Q	G	No	L	V	A	S	No	E	ESEV	E	D
Chicken/New York/13142-5/94 (H7N2)	PEIPKGR	Q	G	No	L	V	A	S	No	E	ESEV	E	D

^*^The H7N2 isolates in this study.

^‡^H3 numbering.

**Table 3:** Virus yield in cloacal and oropharyngeal swabs from chickens.

Virues	Days post-inoculation (log_10_TCID_50_/ml) ± SD^a^							
	3day		5day		7day		10day	
	Oropharyngeal swabs	Cloacal swabs	Oropharyngeal swabs	Cloacal swabs	Oropharyngeal swabs	Cloacal swabs	Oropharyngeal swabs	Cloacal swabs
Maduo06	1.25 (1/10)	ND^b^ (0/10)	3.44 ± 0.58 (4/10)	1.67 ± 0.47 (2/10)	ND (0/10)	ND (0/10)	ND (0/10)	ND (0/10)
Maduo09	2.50 ± 0.69 (3/10)	1.25 (1/10)	1.25 (1/10)	ND (0/10)	ND (0/10)	ND (0/10)	ND (0/10)	ND (0/10)

Maduo06: A/Pika/QH-Maduo/01/2006(H7N2); Maduo09: A/Pika/Maduo/01/2009(H7N2).

^a^For statistical purposes, a value of 1.5 was assigned if virus was not detected from the undiluted sample in three embryonated hen’s eggs.

^b^Not detected.
