# Supplementary material for: Characterization of H7N2 Avian Influenza Virus in Wild Birds and Pikas in Qinghai-Tibet Plateau Area
Source: Sci Rep. 2016 Aug 24;6:30974. doi: 10.1038/srep30974 (PMC4995509; doi:10.1038/srep30974)
Supplement: Supplementary Information [file srep30974-s1.doc]

**Supplementary Material**

**Characterization of H7N2 Avian Influenza Virus in Wild Birds and Pikas in Qinghai-Tibet Plateau Area**

Shuo Su^1^, Gang Xing ^1,2^, Junhua Wang^2^, Zengkui Li^4^, Jinyan Gu^1^, Liping Yan^1^, Jing Lei^1^, Senlin Ji^1^, Boli Hu^1^, Gregory C. Gray^5^, Yan Yan^2^, Jiyong Zhou^1,2,3,*^

^1^Jiangsu Engineering Laboratory of Animal Immunology, Institute of Immunology and College of Veterinary Medicine, Nanjing Agricultural University, Nanjing, China

^2^Key Laboratory of Animal Virology of Ministry of Agriculture, College of Animal Sciences, Zhejiang University, Hangzhou, China

^3^Collaborative Innovation Center and State Key Laboratory for Diagnosis and Treatment of Infectious Diseases, The First Affiliated Hospital, Zhejiang University, Hangzhou, China

^4^College of Agriculture and Animal Husbandry, Qinghai University, Xining, China

^5^Division of Infectious Diseases, Global Health Institute, & Nicholas School of the Environment, Duke University, Durham, NC, USA

*Corresponding authors:

Jiyong Zhou email : [jyzhou@njau.edu.cn](mailto:jyzhou@zju.edu.cn)

**Figure S1**. Phylogenetic analysis of the NA, PB2, PB1, PA, NP, M, NS genes of the H7N2 isolates. Viruses isolated in this study are indicated in red. Maximum likelihood phylogenetic trees were inferred and with 1000 times bootstrap using MEGA (Version 7).

**NA**

**PB2**

**PB1**

**PA**

**PA**

**M**

**NS**
